# SNX1 inhibits human ovarian cancer progression via regulation of the cell cycle, apoptosis and migration

**DOI:** 10.1080/23723556.2025.2604899

**Published:** 2025-12-31

**Authors:** Pin Li, Xiaoyan Yu, Kailing Wen, Lei Wang, Jingran Yu, Ping Chen

**Affiliations:** a The International Peace Maternity and Child Health Hospital, School of Medicine, Shanghai Jiao Tong University, Shanghai, People's Republic of China; b Shanghai Key Laboratory of Embryo Original Diseases, Shanghai, People's Republic of China; c State Key Laboratory of Systems Medicine for Cancer, Shanghai Cancer Institute, Renji Hospital, School of Medicine, Shanghai Jiao Tong University, Shanghai, People's Republic of China

**Keywords:** SNX1, ovarian cancer, epithelial-mesenchymal transition, paclitaxel, drug sensitivity

## Abstract

Sorting nexin 1 (SNX1), a member of the sorting nexin family, has been implicated in various cellular processes, yet its role in ovarian cancer (OV) remains poorly characterized. In this study, we systematically investigated the expression pattern, prognostic relevance and functional impact of SNX1 in OV. Bioinformatics analysis revealed that SNX1 is significantly downregulated in OV tissues, and its low expression is associated with poor overall and progression-free survival. Gene set enrichment analysis indicated that SNX1 downregulation is linked to activation of cancer-related pathways, including p53 signaling, PI3K/AKT signaling, and cell cycle–associated programs such as E2F targets and G2/M checkpoint. Functionally, SNX1 overexpression inhibited OV cell proliferation, blocked G1/S transition (with downregulation of E2F1, CDK2, CDK6, and cyclin D1), promoted apoptosis, and suppressed cell migration by modulating EMT markers (upregulating E-cadherin; downregulating *N*-cadherin, vimentin, Snail1, and *β*-catenin). Drug sensitivity analysis demonstrated a synergistic anti-tumor effect between SNX1 overexpression and paclitaxel treatment. Collectively, our findings identify SNX1 as a tumor suppressor and potential therapeutic target in OV, functioning through regulation of cell cycle, apoptosis and migration.

## 1. Introduction

Ovarian cancer (OV) is one of the most lethal gynecological malignancies, primarily due to its asymptomatic onset and late-stage diagnosis, resulting in a five-year survival rate of less than 50%.^1^^,^^2^ Although advances in cytoreductive surgery and platinum-based chemotherapy have improved initial response rates, the majority of patients eventually relapse and develop chemoresistance, which remains a major clinical challenge.^3^^,^^4^ The progression and recurrence of OV are driven by a complex interplay of molecular alterations, including dysregulated signaling pathways, epithelial-mesenchymal transition (EMT), immune evasion, and remodeling of the tumor microenvironment (TME).^5-9^ These factors not only promote tumor growth and metastasis but also impair the efficacy of emerging immunotherapies and targeted agents. Therefore, identifying novel regulators that influence these oncogenic processes is essential for improving prognostic evaluation and therapeutic strategies in OV.

Sorting nexins (SNXs) are a conserved family of proteins involved in endosomal trafficking, signal transduction, and membrane remodeling.^10^ Several members of the SNX family have been implicated in cancer development through modulation of receptor recycling, growth signaling, and intracellular transport.^11-14^ Among them, sorting nexin 1 (SNX1) was first identified as a protein interacting with the epidermal growth factor receptor (EGFR) and has since been reported to act as a tumor suppressor in various cancers, including lung, gastric, and colorectal cancers.^15-19^ However, the role of SNX1 in ovarian cancer remains largely unexplored. Given its known regulatory functions and tumor-suppressive potential, investigating the expression pattern, functional significance, and underlying mechanisms of SNX1 in OV may uncover new insights into disease pathogenesis and offer opportunities for therapeutic intervention.

In this study, we revealed that SNX1 is significantly downregulated in ovarian cancer and correlates with poor prognosis and reduced sensitivity to chemotherapeutic agents such as paclitaxel, docetaxel, and 5-fluorouracil. Functional assays showed that SNX1 overexpression suppresses cell proliferation via G1/S arrest, promotes apoptosis, and inhibits EMT-related migration. Enrichment further linked low SNX1 expression to activation of oncogenic pathways. These findings highlight SNX1 as a potential tumor suppressor, with promise as a prognostic marker and therapeutic target in OV.

## 2. Materials and methods

### 2.1. Data sources and processing

Transcriptomic data and corresponding clinical information were obtained from multiple public databases. Expression profiles of OV and normal ovarian epithelial tissues were downloaded from The Cancer Genome Atlas (TCGA) and the Genotype-Tissue Expression (GTEx) project via the UCSC Xena platform (https://xena.ucsc.edu/). Additionally, microarray datasets GSE38666, GSE40595, and GSE105437 were retrieved from the Gene Expression Omnibus (GEO) database. For TCGA and GTEx datasets, expression values were transformed to log₂ (TPM + 1) to ensure cross-dataset comparability. GEO datasets were processed using the R package GEOquery (v2.74.0), including background correction, normalization, and batch effect correction. To characterize the broader expression pattern of the SNX family, pan-cancer analysis was conducted using transcriptomic profiles of 9,018 tumor and 8,160 normal tissue samples across 30 cancer types. The mRNA expression levels of all SNX family members were compared between tumor and matched normal tissues.

### 2.2. Expression and survival analysis

SNX1 expression levels were analyzed across TCGA, GTEx, and GEO datasets. The Wilcoxon rank-sum test was used to compare SNX1 expression between OV tumors (*n* = 421 from TCGA) and normal ovarian tissues (*n* = 88 from GTEx).

Survival analysis was performed using the TCGA-OV cohort. Patients were stratified into high and low SNX1 expression groups based on the median expression value. Kaplan-Meier survival curves were generated to assess the association between SNX1 expression and overall survival (OS; *n* = 1530) and progression-free survival (PFS; *n* = 1338), with statistical significance evaluated using the log-rank test.^20^

### 2.3. Gene set enrichment analysis (GSEA)

To explore the biological pathways associated with SNX1 expression, TCGA-OV samples were divided into SNX1-high and SNX1-low groups based on median expression. GSEA was performed using the R package clusterProfiler (v4.10.1), with gene sets sourced from the MSigDB Hallmark and KEGG databases. Normalized enrichment scores (NES) and adjusted *p*-values (Benjamini–Hochberg correction) were calculated. Pathways with NES > 1 and *p.adjust* < 0.05 were considered significantly enriched and visualized using bar plot.

### 2.4. Chemotherapy response prediction

Drug sensitivity analysis was conducted using the R package oncoPredict to predict response to commonly used chemotherapeutic agents in OV, including 5-fluorouracil, docetaxel, paclitaxel, cisplatin and oxaliplatin, based on SNX1 expression profiles.^21^

### 2.5. Cell culture and rationale for cell line selection

To comprehensively investigate the functional role of SNX1 in OV, we employed a panel of well-characterized human OV cell lines representing diverse histopathological subtypes, genetic backgrounds, and phenotypic features to enhance the biological relevance and generalizability of our findings. These included A2780, a cisplatin-sensitive epithelial ovarian cancer cell line originally derived from an untreated patient with endometrioid ovarian carcinoma, widely used for studying chemosensitivity and drug response; HEY, established from a moderately differentiated papillary serous cystadenocarcinoma, commonly used to model serous ovarian cancer with moderate invasiveness and epithelial morphology; SKOV3, derived from the ascites of a patient with adenocarcinoma, serving as a high-grade serous carcinoma model characterized by aggressive growth and intrinsic platinum resistance, carrying a homozygous TP53 deletion; CAOV-3, a high-grade serous ovarian carcinoma cell line harboring TP53 mutations and moderate chemotherapy sensitivity, genetically representative of clinical high-grade serous ovarian cancer (HGSOC); OVCAR-8, established from a patient with papillary serous adenocarcinoma, exhibiting strong cisplatin resistance and widely used as a chemoresistant model; COV362, a high-grade serous ovarian cancer line characterized by BRCA1 mutation and TP53 alteration, derived from a chemotherapy-naïve patient and relevant for BRCA-related research; TOV21G, originating from primary ovarian clear cell carcinoma (OCCC) harboring ArId1A mutations and typical clear cell features, valuable for subtype-specific studies; and HFTEC (human fallopian tube epithelial cells), used as non-malignant controls representing the proposed cell-of-origin for high-grade serous ovarian carcinoma, thus providing a physiologically relevant baseline for comparison.

For functional experiments, A2780 and HEY cell lines were specifically chosen for SNX1 overexpression assays. Western blot assays revealed that these two cell lines had the lowest endogenous SNX1 expression among the tested models, which is consistent with the observed downregulation of SNX1 in clinical ovarian cancer samples. Using low-SNX1–expressing lines allowed us to simulate the pathological state and more accurately evaluate the phenotypic impact of SNX1 restoration.

Human-derived ovarian cancer cell lines (SKOV3, A2780, CAOV-3, HEY, OVCAR-8, COV362, TOV21G) and human fallopian tube epithelial cells (HFTEC) were purchased from the American Type Culture Collection (ATCC, Manassas, VA, U.S.A). A2780, HEY, CAOV-3, TOV-21G, COV362, HEK-293T, and HFTEC cells were cultured in high-glucose DMEM (BasalMedia). SKOV3 cells were maintained in McCoy’s 5 A medium (BasalMedia), while OVCAR-8 was grown in RPMI-1640 medium (BasalMedia). All culture media were supplemented with 10% fetal bovine serum (FBS) and 1% penicillin-streptomycin (Pen-Strep). All cells were maintained at 37 °C in a humidified incubator with 5% CO₂ and preserved at the Shanghai Cancer Institute.

### 2.6. Lentiviral packaging, transfection, and puromycin selection

Full-length human SNX1 cDNA was cloned into the pLX304-Blast-V5 vector (Addgene #25890) downstream of the EF-1α promoter to generate the SNX1-OE construct. The corresponding empty vector (SNX1-vector) lacking the SNX1 insert served as the negative control. Both constructs include a puromycin resistance gene and a C-terminal V5 epitope tag, enabling selection and detection. This isogenic pair was used to control for vector backbone, selection pressure, and potential effects of the V5 tag.

For lentiviral packaging, SNX1-OE or SNX1-vector plasmids were co-transfected with the packaging plasmids psPAX2 and pMD2.G into HEK-293T cells using jetPRIME® Polyplus transfection reagent (Polyplus), following the manufacturer’s protocol. After 48 hours of incubation at 37 °C in a humidified 5% CO₂ incubator, the culture supernatant containing lentiviral particles was harvested, passed through a 0.45 μm filter to remove cell debris, and either used immediately or stored at −80 °C.

To generate stably transduced cell lines, ovarian cancer cells were seeded in 6-well plates and cultured overnight to reach 30–50% confluence. The filtered lentiviral supernatant, supplemented with 8 μg/mL polybrene (Sigma-Aldrich), was added to the cells. Plates were centrifuged at 1,200 × g for 1 hour to enhance transduction efficiency, followed by a 24-hour incubation at 37 °C with 5% CO₂. The medium was then replaced with fresh complete culture medium.

At 48 hours post-transduction, 2 μg/mL puromycin (InvivoGen) was added to the culture medium to initiate selection. Cells were maintained under puromycin selection for 5–7 d, with medium changes every 2–3 d. Successfully transduced cells were identified by puromycin resistance and subsequently validated for SNX1 overexpression by western blot assays before use in downstream experiments.

### 2.7. Western blot assay

Cells were lysed using a protein extraction kit (SB-BR040, Share-Bio). The supernatant was collected, and protein concentration was quantified by BCA assay (P0011, Beyotime). Equal amounts of protein (30 µg) were separated by 10% SDS-PAGE (G2043-50T, Servicebio) and transferred to nitrocellulose membranes (Millipore).

After blocking with 5% skimmed milk, the membranes were incubated overnight at 4 °C with the following primary antibodies diluted in TBST containing 1% BSA: anti-beta-Actin (20536-1-AP, 1:1000, Proteintech), anti-SNX1 (14914-1-AP, 1:1000, Proteintech), anti-CDK2 (10122-1-AP, 1:1000, Proteintech), anti-CDK6 (14052-1-AP, 1:1000, Proteintech), anti-Cyclin D1 (60186-1-AP, 1:1000, Proteintech), anti-E2F1 (12171-1-AP, 1:1000, Proteintech), anti-GAPDH (60004-1-Ig, 1:5000, Proteintech), followed by 1-hour incubation with corresponding HRP-conjugated secondary antibodies (Goat Anti-Rabbit: HA1001 or Goat Anti-Mouse: HA1006, 1:10000, Huabio) at room temperature. Protein bands were visualized using an ECL kit (P0018S, Beyotime) and imaged with a chemiluminescence system (Bio-Rad, U.S.A.). Band intensities were quantified using ImageJ software and normalized to *β*-actin or GAPDH. All experiments were independently repeated three times, and quantitative data are shown in supplementary figures.

### 2.8. Cell viability assay

Cell viability was assessed using the Cell Counting Kit-8 (C0037, Beyotime). A2780 and HEY cells were seeded into 96-well plates at a density of 2,000 cells per well in 100 µL of complete medium and incubated overnight at 37 °C with 5% CO₂. At 0, 24, 48, 72, and 96 hours, 10 µL of CCK-8 reagent was added per well and incubated for 2 hours at 37 °C. Absorbance was measured at 450 nm using a Synergy Neo2 Hybrid microplate reader (Agilent). For drug treatment assays, cells were treated with either carboplatin (MCE, HY-17393) or paclitaxel (MCE, HY-B0015) for 24 hours. Each experimental condition included five technical replicates per group and was independently repeated three times. Mean absorbance values were used for statistical analysis.

### 2.9. 5‐ethynyl‐2′‐deoxyuridine staining assay

Cells were seeded in µ-Slide 8-Well chambers (80826, ibidi GmbH) at a density of 20,000 cells per well and incubated overnight. DNA synthesis was evaluated using the 5‐ethynyl‐2′‐deoxyuridine (EdU) Cell Proliferation Kit with Alexa Fluor 488 (C0071S, Beyotime). EdU (10 µM) was added to fresh medium and incubated for 2 hours at 37 °C. Cells were then fixed with 4% paraformaldehyde, permeabilized with 0.3% Triton X-100, and stained using the Click-iT reaction cocktail. Nuclei were counterstained with DAPI (1 µg/mL, Beyotime). Fluorescence images were acquired using a Leica confocal microscope. The percentage of EdU-positive nuclei was calculated using ImageJ software. For chemotherapeutic response analysis, A2780 and HEY cells were treated with paclitaxel (MCE, HY-B0015) for 24 hours prior to EdU labeling to assess proliferation under drug stress. Quantification was based on five randomly selected fields per group, and the assay was independently repeated three times.

### 2.10. Cell cycle assay

Cell cycle analysis were performed using the BeyoClick™ EdU Cell Proliferation Kit with Alexa Fluor 488 (Beyotime, C0071S) according to the manufacture’s instruction. Briefly, cells were pulsed with 10 μM EdU for 2 hours. Then cells were harvested, washed with PBS (containing 3% BSA), and and fixed in 4% paraformaldehyde. After fixation, cells were incubated with RNase A for 30 min at 37 °C, and stained with 50 μg/mL propidium iodide (PI) for 30 min. Flow cytometry was performed using a FACS Calibur instrument (BD Biosciences), and data were analyzed with CellQuest and FlowJo software to determine the proportions of cells in G1, S, and G2/M phases. Each condition was analyzed using three technical replicates, and the experiment was repeated independently three times.

### 2.11. Quantitative real-time PCR (qRT-PCR) assay

Total RNA was extracted with TRIzol reagent (15596-026, Noble Ryder) and quantified using NanoDrop spectrophotometer (Thermo Fisher Scientific, U.S.A). cDNA was synthesized from 1 μg RNA using a cDNA synthesis kit(SB-RT001, Share-Bio). QPCR was performed using 2 × Fast SYBR Green qPCR Master Mix (G3323-1, Servicebio) on a 7500 Real-Time PCR System (Thermo Fisher Scientific) with the following protocol: initial denaturation, 40 amplification cycles, and melt curve analysis. The relative mRNA expression of target genes was calculated by the 2^^-ΔΔCT^ method using 18S rRNA and ACTB as reference genes. Primer sequences are listed in Table 1.

**Table 1.** Primer sequences for qRT-PCR assays are listed below.

Primer	Forward (5’→3’)	Reverse (5’→3’)
18 s rRNA	TGCGAGTACTCAACACCAACA	GCATATCTTCGGCCCACA
*ACTB*	CATGTACGTTGCTATCCAGGC	CTCCTTAATGTCACGCACGAT
*CDH1*	GGCTGGACCGAGAGAGTTTC	CGACGTTAGCCTCGTTCTCA
*CDH2*	AGGCTTCTGGTGAAATCGCA	TGCAGTTGCTAAACTTCACATTG
*VIM*	CTCCCTGAACCTGAGGGAAAC	TTGCGCTCCTGAAAAACTGC
*SNAI1*	TAGCGAGTGGTTCTTCTGCG	TGCTGGAAGGTAAACTCTGGA
*CTNNB1*	GAGGAGCAGCTTCAGTCCC	TCCAACTCCATCAAATCAGCTTG

### 2.12. Migration assay

Cell migration ability was evaluated using an automated wound-healing (scratch) assay. A2780 and HEY cells were seeded in 24-well plates at a density of 50,000 cells per well and allowed to reach confluence. A linear scratch was generated using a 200-μL pipette tip. Detached cells were removed by washing with PBS, and fresh complete medium was added. Migration assays were repeated using a live-cell imaging workstation (Olympus IX83). Images of the scratch area were automatically captured every 6 hours for 48 hours under controlled incubation conditions. To quantify cell migration objectively, we implemented a standardized ImageJ (v1.54) analysis workflow:

1) Preprocessing: Images were converted to 8-bit grayscale; contrast was enhanced (Process → Enhance Contrast, saturated pixels set to 0.5%);
2) Edge Detection: Applied smoothing (Process → Smooth) and edge detection (Process → Find Edges);
3) Thresholding: Binary images of cell-free scratch areas were generated (Image → Adjust → Threshold);
4) Measurement: The initial wound area (time 0 h) and corresponding areas at subsequent time points were measured using the wand tool.

The migration rate (%) was calculated as: Migration rate = 100% * (Initial scratch area - Scratch area at time *t*)/Initial scratch area. Three biological replicates were performed for each condition, and representative images are presented in the updated Figures.

### 2.13. Apoptosis assay

Apoptosis was evaluated using the Annexin V-647A Apoptosis Detection Kit (SB-Y6026, Share-Bio). Cells were seeded into 6-well plates (500,000 cells/well) and cultured for 24 hours. Subsequently, medium was replaced with serum-free DMEM, and cells were incubated for an additional 24 hours to induce apoptosis. Cells were harvested, washed with PBS, and stained with Annexin V-647A for 15 minutes at room temperature in the dark, following the manufacturer’s protocol. Flow cytometry was performed using a FACS Calibur system (BD Biosciences). Data were analyzed based on Annexin V and propidium iodide (PI) staining to determine the percentage of early and late apoptotic cells. Apoptosis assays were independently repeated three times for each experimental group.

### 2.14. Statistical analysis

Statistical analyzes were performed using GraphPad Prism (version 9.0) and R software (version 4.2.3). Data are presented as mean ± standard deviation (SD). The two-tailed Student's *t*-test was used to evaluate statistical differences between two groups, while one-way analysis of variance (ANOVA) was applied for comparisons among multiple groups. Two-way ANOVA was performed when assessing the interaction between two independent variables. A *p*-value < 0.05 was considered statistically significant. All experiments were repeated at least three times to ensure data reliability and reproducibility.

## 3. Results

### 3.1. SNX1 downregulation is associated with poor prognosis in ovarian cancer

To identify sorting SNX family members with potential clinical relevance in OV, a pan-cancer expression analysis was conducted using TCGA and GTEx datasets. Among 33 SNX genes evaluated across 30 tumor types, OV displayed a striking pattern, with 21 SNX genes significantly downregulated in tumor tissues compared to normal controls (Supplementary Figure 1A). Survival analysis within the TCGA-OV cohort revealed that reduced expression of SNX1, SNX2, and SNX25 was significantly correlated with poorer overall survival (Supplementary Figure 1B). It is important to note that the TCGA and GTEx datasets used in this pan-cancer analysis lack annotation for ovarian cancer histological subtypes (Type I and Type II), thus the observed downregulation could not be stratified by subtype at this stage. Given SNX1’s previously reported tumor-suppressive role in colorectal cancer and its consistent downregulation in OV, SNX1 was selected for further investigation.

**Figure 1.**
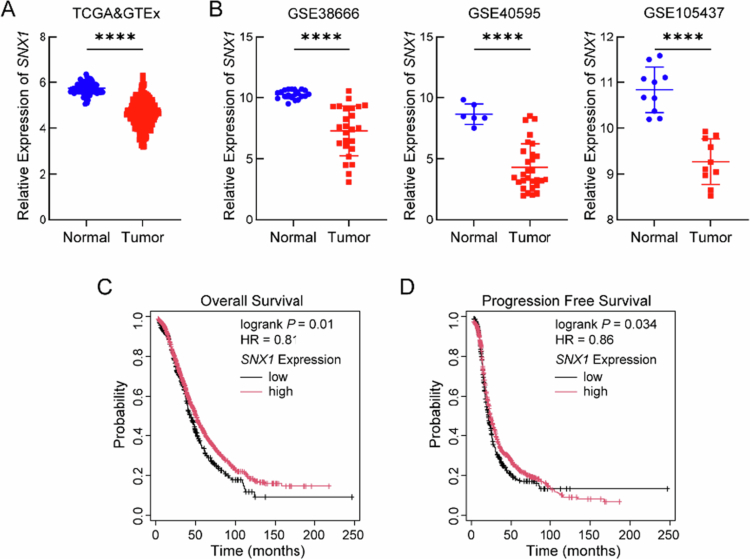
SNX1 downregulation is associated with poor prognosis in OV. (A) SNX1 mRNA expression in OV tissues versus normal ovary tissues from the TCGA and GTEx databases (****P < 0.0001, vs. healthy ovarian tissues, Wilcoxon test). (B) SNX1 mRNA expression in OV tissues and normal tissues from the GSE38666, GSE40595, and GSE105437 datasets (****P < 0.0001, vs. healthy ovarian tissues, Wilcoxon test). (C) Kaplan–Meier analysis of overall survival based on SNX1 expression in the TCGA-OV cohort (*P* = 0.01, vs. SNX1-low expression groups, log-rank test). (D) Kaplan–Meier analysis of progression-free survival based on SNX1 expression in the TCGA-OV cohort (*P* = 0.034, vs. SNX1-low expression groups, log-rank test).

Analysis of the TCGA (421 tumor samples) and GTEx (88 normal samples) datasets showed that SNX1 expression was significantly lower in OV tissues relative to normal ovarian tissues (*P* < 0.05) (Figure 1A). This downregulation was further validated in three independent GEO datasets, all of which consistently demonstrated reduced SNX1 expression in OV samples (*P* < 0.05) (Figure 1B). Kaplan–Meier survival analysis based on 1530 patients for overall survival and 1338 patients for progression-free survival indicated that low SNX1 expression was significantly associated with both shorter overall survival and progression-free survival in OV patients (*P* < 0.05) (Figure 1C-D). These findings suggest that SNX1 downregulation is a frequent event in OV and may serve as a potential prognostic biomarker associated with unfavorable clinical outcomes.

### 3.2. SNX1 expression is associated with dysregulation of cell cycle and oncogenic signaling pathways

**Figure 2.**
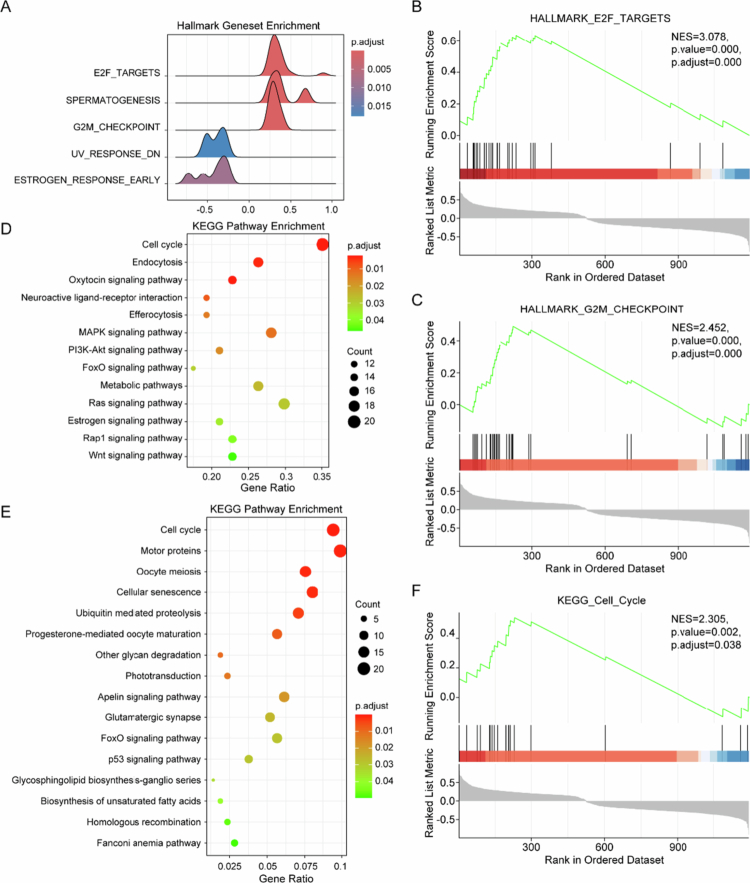
SNX1 expression is associated with dysregulation of cell cycle and oncogenic signaling pathways. (A) Hallmark gene sets enriched in the SNX1-low expression group based on GSEA of TCGA-OV data, ranked by adjusted *p*-value. (B) GSEA enrichment plot of the E2F target gene set in the SNX1-low expression group (*P* < 0.001). (C) GSEA enrichment plot of the G2/M checkpoint gene set in the SNX1-low expression group (*P* < 0.001). (D) KEGG pathways enriched in the SNX1-low expression group based on GSEA, ranked by adjusted *p*-value. (E) KEGG pathways identified by classical over-representation analysis in the SNX1-low expression group, ranked by adjusted *p*-value. (F) GSEA enrichment plot of the cell cycle pathway in the SNX1-low expression group (*P* = 0.002).

To investigate the molecular pathways associated with SNX1 expression in ovarian cancer, transcriptomic data from the TCGA-OV cohort were analyzed. Gene set enrichment analysis using Hallmark gene sets revealed that low SNX1 expression was significantly associated with the upregulation of cell cycle–related pathways, particularly E2F targets and the G2/M checkpoint (Figure 2A–C). Complementary KEGG pathway enrichment analyzes, performed using both GSEA and classical over-representation methods, consistently demonstrated enrichment of cell cycle signaling in the SNX1-low-expression group (Figure 2D–F). In addition to cell cycle–associated pathways, several hallmark oncogenic signaling pathways—including PI3K-Akt, MAPK, and p53 signaling—also showed significant enrichment in the SNX1-low group across both analytical approaches. These results suggest that reduced SNX1 expression is linked to dysregulated cell cycle control and aberrant activation of key cancer-related signaling cascades in ovarian cancer. The consistent enrichment of these pathways supports a putative regulatory role for SNX1 and provides a basis for further investigation into its mechanistic involvement in tumor progression.

### 3.3. SNX1 suppresses OV cell proliferation via cell cycle regulation

**Figure 3.**
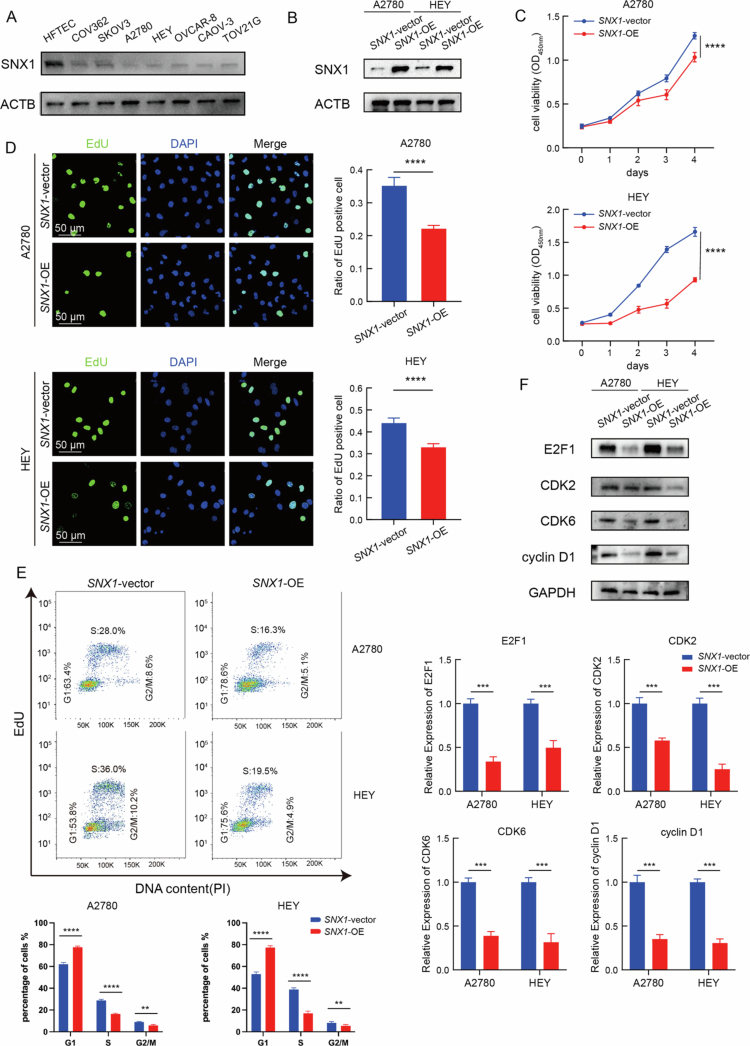
SNX1 suppresses OV cell proliferation via cell cycle regulation. (A) Western blot assays of endogenous SNX1 protein levels in OV cell lines and a normal fallopian tube epithelial cell line. (B) Validation of SNX1 overexpression in A2780 (left) and HEY (right) cells by western blot assays. (C) Cell viability assays of SNX1-overexpressing and control groups in A2780 (top) and HEY (bottom) cells detected by CCK-8 assays (****P < 0.0001, *n* = 5, vs. SNX1-vector groups, two-way ANOVA). (D) Representative EdU staining images (left) and quantification of proliferating cells (right) in SNX1-overexpressing and control groups in A2780 (top) and HEY (bottom) cells. Green: EdU; blue: DAPI. Scale bar, 50 μm. (****P < 0.0001, *n* = 5, vs. SNX1-vector groups, Student’s *t*-test). (E) EdU/PI Flow cytometric analysis of cell cycle distribution in SNX1-overexpressing and control groups in A2780 (top) and HEY (bottom) cells (***P < 0.001, ****P < 0.0001, *n* = 3, SNX1-OE groups vs. SNX1-vector groups, two-way ANOVA). (F) Western blot assays and quantitative analysis of western blot images from three independent biological replicates of E2F1, CDK2, CDK6, and cyclin D1 protein levels in SNX1-overexpressing and control groups in A2780 (left) and HEY (right) cells.

SNX1 expression was first assessed in a panel of human OV cell lines and compared to a normal fallopian tube epithelial cell line. Western blot assays revealed reduced SNX1 protein levels in multiple OV cell lines, with particularly low expression observed in A2780 and HEY cells (Figure 3A, Supplementary Figure 2A). These two cell lines were selected for subsequent gain-of-function studies. Lentivirus-mediated overexpression of SNX1 was confirmed by western blot assays, indicating markedly increased SNX1 levels in both A2780 and HEY cells compared to vector controls (Figure 3B, Supplementary Figure 2B).

To determine the functional consequences of SNX1 overexpression, cell proliferation was evaluated. CCK-8 assays showed significantly decreased cell viability in SNX1-overexpressing A2780 and HEY cells (*P* < 0.05) (Figure 3C). Similarly, EdU incorporation assays revealed reduced percentages of EdU-positive cells in the overexpression groups, indicating impaired DNA synthesis and cell proliferation (Figure 3D). Given the observed enrichment of cell cycle pathways in SNX1-low tumors (Figure 2), cell cycle distribution was assessed by EdU/PI flow cytometry. SNX1-overexpressing cells exhibited a decrease in S phase and a corresponding reduction in G2/M phase (Figure 3E), suggesting that SNX1 induces a block at the G1/S transition. To further investigate the underlying mechanism, protein levels of key cell cycle regulators were examined. Western blot assays revealed decreased expression of CDK2, CDK6, cyclin D1 (CCND1), and E2F1 in the SNX1-overexpressing groups (Figure 3F, Supplementary Figure 2C), implicating suppression of E2F signaling as a potential mechanism contributing to cell cycle arrest.

To evaluate whether the tumor-suppressive effects of SNX1 could be generalized, particularly in the context of the prevalent p53-mutant high-grade serous ovarian carcinoma (HGSOC), we extended our analysis to the CAOV-3 cell line, which harbors a p53 mutation. Consistent with the findings in A2780 and HEY cells, SNX1 overexpression in CAOV-3 cells significantly inhibited cell viability (Supplementary Figure 2F), slowed colony formation(Supplementary Figure 2I) and induced S-phase arrest with a concomitant reduction in G2/M phase as assessed by EdU/PI flow cytometry (Supplementary Figure 2H). Furthermore, Western blot analysis confirmed a decrease in E2F1 expression upon SNX1 overexpression in this model (Supplementary Figure 2G).

These findings demonstrate that SNX1 overexpression inhibits OV cell proliferation by inducing S-phase arrest and downregulating components of the E2F pathway. Together with transcriptomic data, the results support a role for SNX1 as a negative regulator of ovarian cancer cell growth, potentially through modulation of cell cycle progression, and indicate that this function is conserved in a p53-mutant HGSOC background.

### 3.4. SNX1 promotes apoptosis and suppresses migration in OV

**Figure 4.**
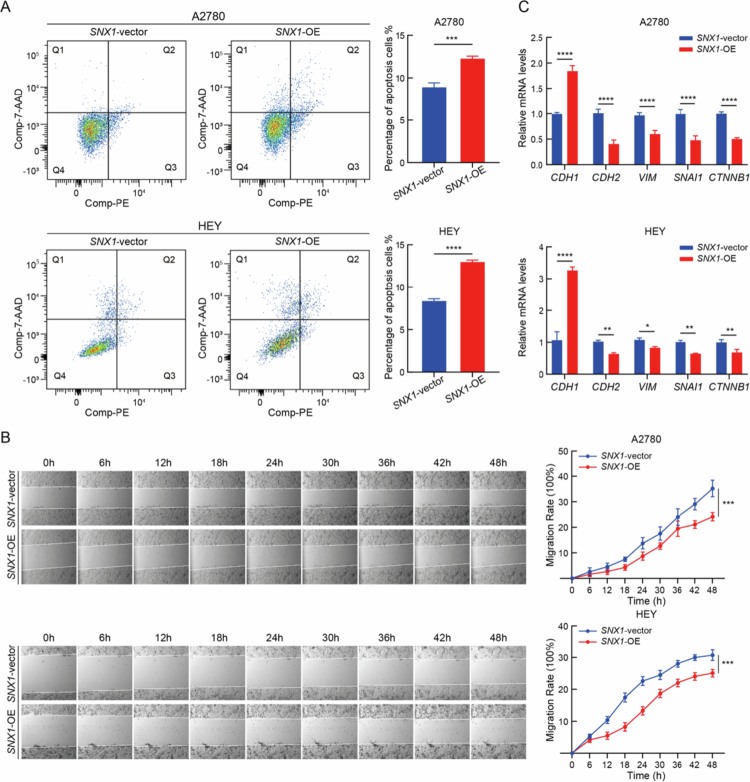
SNX1 promotes apoptosis and suppresses migration in OV. (A) Apoptosis levels induced by serum starvation in SNX1-overexpressing and control groups in A2780 (top) and HEY (bottom) cells, measured by flow cytometry assays (***P < 0.001, ****P < 0.0001, *n* = 3, vs. SNX1-vector groups, Student’s *t*-test). (B) Representative images (left) and quantification (right) of wound healing assays in SNX1-overexpressing and control groups in A2780 (top) and HEY (bottom) cells (****P < 0.001, *n* = 6, vs. SNX1-vector groups, two-way ANOVA). (C) mRNA expression levels of CDH1, CDH2, VIM, SNAI1, and CTNNB1 in SNX1-overexpressing and control groups in A2780 (top) and HEY (bottom) cells, detected by qRT-PCR assays (*P < 0.05, **P < 0.01, ****P < 0.0001, *n* = 3, vs. SNX1-vector groups, Student’s *t*-test).

The functional role of SNX1 in ovarian cancer progression was further assessed using A2780 and HEY cell lines with SNX1 overexpression. To induce apoptosis, cells were subjected to serum starvation. Flow cytometry analysis revealed a significant increase in apoptotic cell populations in both cell lines compared to vector controls, indicating a pro-apoptotic effect associated with elevated SNX1 expression (*P* < 0.05) (Figure 4A).

To evaluate the influence of SNX1 on cell migration, scratch wound healing assays were performed. A marked reduction in wound closure was observed in SNX1-overexpressing cells, suggesting a decreased migratory capacity (*P* < 0.05) (Figure 4B). Additionally, quantitative PCR analysis was conducted to examine the expression of epithelial–mesenchymal transition (EMT) markers. SNX1 overexpression led to increased expression of CDH1 (E-cadherin) and decreased levels of CDH2 (*N*-cadherin), VIM (vimentin), SNAI1 (Snail1), and CTNNB1 (*β*-catenin) (*P* < 0.05), consistent with EMT suppression (Figure 4C). Collectively, these findings suggest that SNX1 exerts tumor-suppressive effects in ovarian cancer by promoting apoptosis and inhibiting migration, potentially through the disruption of EMT-associated pathways. These results further support the therapeutic relevance of SNX1 in limiting ovarian cancer progression.

### 3.5. SNX1 enhances sensitivity to paclitaxel in OV cells

**Figure 5.**
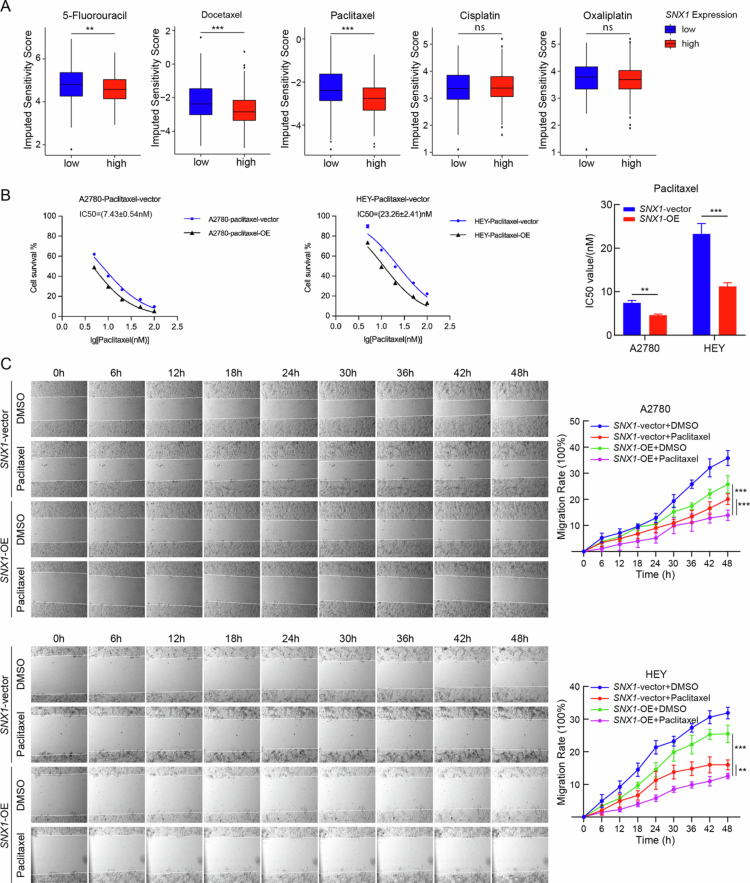
SNX1 enhances sensitivity to paclitaxel in OV cells. (A) Drug sensitivity analysis between SNX1-low and SNX1-high groups using "oncoPredict" R package (**P < 0.01, ***P < 0.001, vs. SNX1-low expression groups, Wilcoxon test). (B) The cell survival of SNX1-overexpressing and control groups in A2780 (left) and HEY (right) cells treated with different concentrations of paclitaxel was assessed using the CCK-8 assays (**P < 0.01, ***P < 0.001, *n* = 5, vs. SNX1-vector groups, Student's *t*-test). (C) Representative scratch wound healing assay images (left) and analysis (right) of SNX1-overexpressing and control groups in A2780 (top; treated with 5 nM paclitaxel or DMSO) and HEY (bottom; treated with 10 nM paclitaxel or DMSO) cells (*P* < 0.01,*P < 0.001, *n* = 6, vs. SNX1-vector groups, two-way ANOVA).

To investigate the potential role of SNX1 in modulating chemosensitivity in ovarian cancer, drug response prediction analysis was performed for multiple chemotherapeutic agents, including 5-fluorouracil, docetaxel, paclitaxel, cisplatin, and oxaliplatin. Based on RNA-seq data from the TCGA-OV cohort, tumors with low SNX1 expression exhibited significantly lower predicted sensitivity (higher IC_50_) to 5-fluorouracil, docetaxel, and paclitaxel, whereas no significant differences were observed for cisplatin or oxaliplatin between SNX1-low and SNX1-high expression groups (Figure 5A). Although the effect sizes were relatively moderate, the consistent and statistically significant differences in predicted sensitivity across multiple agents suggest a potential role for SNX1 in modulating chemotherapy response. These findings provided the rationale for further experimental validation.

To validate these predictions experimentally, A2780 and HEY ovarian cancer cells with or without SNX1 overexpression were treated with paclitaxel and carboplatin. In both cell lines, SNX1 overexpression significantly reduced the IC_50_ of paclitaxel, indicating increased drug sensitivity (Figure 5B). In contrast, SNX1 overexpression did not significantly alter carboplatin sensitivity (Supplementary Figure 3A). Further functional assays were conducted to assess whether SNX1 overexpression could enhance the response to paclitaxel in terms of migration and proliferation. Scratch wound healing assays revealed that the combination of SNX1 overexpression and paclitaxel treatment resulted in significantly greater inhibition of cell migration compared to either treatment alone (Figure 5C). However, EdU incorporation assays did not demonstrate a synergistic effect between SNX1 overexpression and paclitaxel treatment (Supplementary Figure 3B), which may be due to paclitaxel’s primary mechanism targeting microtubule stabilization rather than DNA synthesis. These results suggest that SNX1 overexpression selectively enhances the sensitivity of ovarian cancer cells to paclitaxel but not to carboplatin, highlighting the need for further mechanistic studies to validate SNX1 as a biomarker for differential chemosensitivity in ovarian cancer.

## 4. Discussion

In this study, we identified SNX1 as a potential tumor suppressor in OV, exhibiting a multifaceted role in the regulation of cell proliferation, epithelial–mesenchymal transition and drug sensitivity. Mechanistically, we observed that SNX1 overexpression led to a block at the G1/S transition, accompanied by a notable downregulation of cyclin D1, CDK2, and CDK6. Importantly, this was coupled with the suppression of E2F1, a key transcription factor downstream of the CDK–Rb axis.^22^^,^^23^ Given that CDK-mediated Rb phosphorylation is essential for releasing E2F1 to promote transcription of S-phase genes, our findings suggest that SNX1 inhibits the CDK–Rb–E2F pathway, thereby halting cell cycle progression.

This regulatory axis is frequently dysregulated in OV, where overactive CDK/cyclin complexes and E2F hyperactivation contribute to uncontrolled proliferation and poor prognosis.^24^^,^^25^ Previous studies have shown that elevated CDK2 and CDK6 expression is associated with chemoresistance and poor survival in various cancers.^26-29^ By suppressing both CDKs and E2F1, SNX1 may serve as a key upstream modulator restraining this oncogenic circuit. The mechanism by which SNX1 downregulates CDK–E2F signaling remains to be elucidated. It is possible that SNX1 modulates CDK activity through transcriptional repression, promotion of proteasomal degradation, or by interfering with upstream mitogenic pathways such as the MAPK or PI3K signaling cascades.^30-34^ These potential regulatory routes warrant further investigation.

In addition to cell cycle regulation, our findings demonstrate that SNX1 exerts an inhibitory effect on EMT, as evidenced by the upregulation of epithelial marker E-cadherin and the downregulation of mesenchymal markers such as *N*-cadherin, vimentin, Snail1, and *β*-catenin. EMT is a critical process in OV metastasis, enabling cancer cells to acquire motility and invasive capabilities.^35^^,^^36^ The inverse correlation between SNX1 and mesenchymal markers suggests that SNX1 may function as a metastasis suppressor. Although EMT suppression may in part result from reduced proliferation or S-phase arrest, the concerted modulation of EMT-associated genes indicates a broader transcriptional reprogramming, potentially involving shared regulatory networks between cell cycle and EMT pathways, such as E2F–Snail1 or Wnt/β-catenin signaling.^37-39^

Chemotherapy resistance is a major clinical challenge in OV treatment. Intriguingly, we found that SNX1 overexpression enhanced paclitaxel sensitivity in both A2780 and HEY cells. Paclitaxel primarily targets microtubule dynamics during mitosis, but its efficacy can be influenced by upstream cell cycle regulators and DNA repair mechanisms.^40-42^ The SNX1-mediated suppression of CDK activity may sensitize cells to mitotic stress by interfering with checkpoint adaptation or DNA damage responses.^43^^,^^44^ While the exact molecular link between SNX1 and drug response remains unclear, our results underscore its potential value as a predictive biomarker or therapeutic target to overcome chemoresistance in OV.

It is noteworthy that the phenotypic potency induced by SNX1 overexpression—such as the inhibition of proliferation and the sensitivity to paclitaxel—did not exhibit a simple linear correlation with its protein expression levels in the two ovarian cancer cell lines (A2780 and HEY). This observation suggests that the biological function of SNX1 may be profoundly influenced by cell-type-specific contexts. Inherent genetic variations among different ovarian cancer cell lines may modulate the cellular response threshold to SNX1 signaling or the sensitivity of its downstream effectors. While our functional studies utilized A2780 and HEY cell lines—widely used models for serous ovarian cancer—they do not fully capture the molecular heterogeneity of HGSOC,^45^ such as frequent TP53 or BRCA mutations. To examine whether SNX1 expression differs among ovarian cancer subtypes, we analyzed multiple transcriptomic datasets and observed notable differences between HGSOC and OCCC. These exploratory findings, while not included in the main analysis, suggest potential subtype-specific expression patterns and require further validation in larger, well-annotated cohorts. In addition, clinical metadata such as patient age were not consistently available across datasets. Although we attempted to match normal and tumor tissue types, age-related variability may still act as a confounding factor. These limitations highlight the need for future studies employing more representative and genetically defined models—such as patient-derived organoids or single-cell profiling—to better understand the context-specific role of SNX1 in ovarian cancer.

Beyond SNX1, other sorting nexin (SNX) family members significantly influence cancer progression and therapy response through diverse mechanisms. SNX2 regulates c-Met trafficking and modulates sensitivity to targeted therapies in lung cancer. SNX9 promotes cancer cell invasion and metastasis via endocytic pathways.^46^ Circular SNX25 encodes a protein that enhances DNA damage repair, conferring radioresistance in liver cancer.^47^ In contrast, SNX20 expression predicts a positive response to PD-1 inhibitors in lung adenocarcinoma.^48^ These findings highlight the SNX family's broad impact, suggesting both synergistic and antagonistic roles in cancer biology that warrant further investigation.

In conclusion, our study provides strong evidence that SNX1 functions as a tumor suppressor in ovarian cancer by negatively regulating the CDK–Rb–E2F axis, suppressing EMT and enhancing paclitaxel sensitivity. These multifaceted effects position SNX1 as a promising prognostic biomarker and therapeutic target. Further studies are warranted to elucidate the upstream regulators of SNX1, its downstream effectors, and its therapeutic potential in personalized treatment strategies combining cell cycle inhibitors. Furthermore, investigating the potential crosstalk between SNX1 and other SNX family members in the tumor microenvironment could provide a more comprehensive understanding of the SNX protein network in ovarian cancer pathogenesis and therapy resistance.

## Supplementary Material

PCR_fetal bovine serum.docx PCR_fetal bovine serum.docx

A2780_STR.pdf A2780_STR.pdf

Supplementary Figures.docx Supplementary Figures.docx

CAOV3_STR.pdf CAOV3_STR.pdf

HEY STR.pdf HEY STR.pdf

## Data Availability

The data generated in the present study may be requested from the corresponding author.

## Abbreviations

SNX1: Sorting nexin 1
OV: ovarian cancer
EMT: epithelial-mesenchymal transition
TME: tumor microenvironment
EGFR: epidermal growth factor receptor
TCGA: The Cancer Genome Atlas
GTEx: Genotype Tissue Expression
GEO: Gene Expression Omnibus
OS: overall survival
PFS: progression-free survival
GSEA: gene set enrichment analysis
NES: normalized enrichment scores
