# Supplementary material for: SNX1 inhibits human ovarian cancer progression via regulation of the cell cycle, apoptosis and migration
Source: Mol Cell Oncol. 2025 Dec 31;13(1):2604899. doi: 10.1080/23723556.2025.2604899 (PMC12758353; doi:10.1080/23723556.2025.2604899)
Supplement: PCR_fetal bovine serum.docx [file KMCO_A_2604899_SM0136.docx]

**PCR检测报告**

**报 告 编 号 PCR20250108**

**报 告 名 称 胎牛血清提取DNA PCR检测报告**

**检 测 汇 总**

**SUMMARY**

**检品名称** 胎牛  **动物等级**

**样本类型 血清**   **动物品系**

**检 测 结 果**

**RESULT**

| **样本引物检测结果汇总** | | | |
| --- | --- | --- | --- |
| **检测项目**  **TEST ARTICLES** | **检测依据**  **STANDARD** | **方法**  **METHOD** | **结果**  **RESULT** |
| **金黄色葡萄球菌(SAU)** | **PNK-TW001** | **PCR** | 阴性 |
| **小肠结肠炎耶尔森菌(YEN)** | **PNK-TW002** | **PCR** | 阴性 |
| **小鼠细小病毒(MVM)** | **T/CALAS 28-2017** | **PCR** | 阴性 |
| **沙门氏菌(SE)** | **PNK-TW004** | **PCR** | 阴性 |
| **牛棒状杆菌(C.bovis)** | **T/CALAS 20-2017** | **PCR** | 阴性 |
| **鲍特杆菌(BT)** | **PNK-TW006** | **PCR** | 阴性 |
| **肺炎克雷伯杆菌(KPN)** | **PNK-TW007** | **PCR** | 阴性 |
| **绿脓杆菌(PAV)** | **PNK-TW008** | **PCR** | 阴性 |
| **鼠痘病毒(MPV)** | **T/CALAS 44-2017** | **PCR** | 阴性 |
| **支原体(MYCO)** | **T/CALAS 40-2017（肺支原体）** | **PCR** | 阴性 |
| **仙台病毒(SV)** | **T/CALAS 49-2017** | **PCR** | 阴性 |
| **小鼠肝炎病毒(MHV)** | **T/CALAS 25-2017** | **PCR** | 阴性 |
| **诺如病毒(MNV)** | **T/CALAS 22-2017** | **PCR** | 阴性 |
| **呼肠孤病毒(REOV)** | **T/CALAS 50-2017** | **PCR** | 阴性 |
| **小鼠脑脊髓炎病毒(TMEV)** | **T/CALAS 26-2017** | **PCR** | 阴性 |


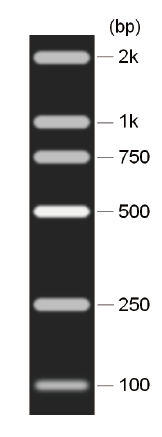
**图1**

**图2**

**
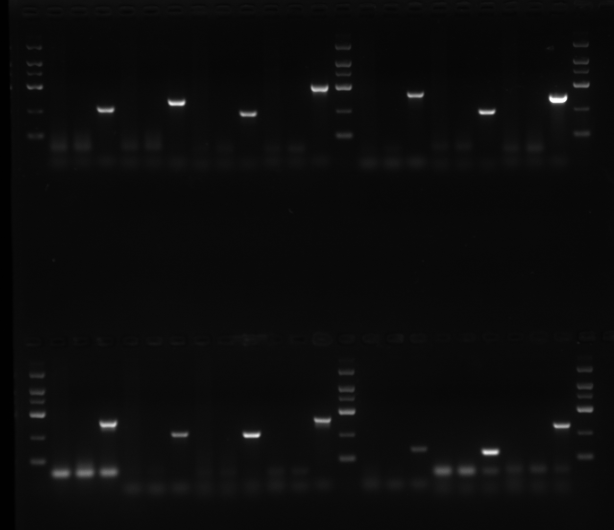
**

FH

阳性

阴性

**
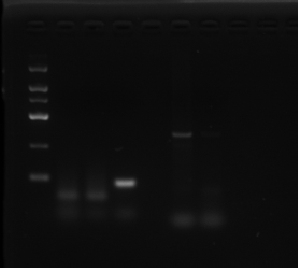
**

FH

GAPDH

DNA cDNA

阳性

阴性

**图3**

图1为电泳所用Marker；图2表格中前14个检测项目，依次为金黄色葡萄球菌，小肠结肠炎耶尔森菌，小鼠细小病毒，沙门氏菌，牛棒状杆菌，鲍特杆菌，肺炎克雷伯杆菌，绿脓杆菌，鼠痘病毒，肺支原体，仙台病毒，小鼠肝炎病毒，诺如病毒和呼肠孤病毒，图3表格中为最后1个检测项目，为小鼠脑脊髓炎病毒检测结果。电泳点样三个为一组，顺序依次为一个待测样品，阴性和阳性对照。

**结论：此次检测如图所示，检测指标均为阴性。**

主检： 朱佳仪 审核：

日期： 2025-01-08 日期:

******报告结束END OF REPORT******
