# Supplementary material for: SNX1 inhibits human ovarian cancer progression via regulation of the cell cycle, apoptosis and migration
Source: Mol Cell Oncol. 2025 Dec 31;13(1):2604899. doi: 10.1080/23723556.2025.2604899 (PMC12758353; doi:10.1080/23723556.2025.2604899)

Assessment of the Quality of Cytogenetic Testing

Cell Line Authentication Service

STR genotype test report

Sample INFORMATION

Sample ID:

Customer Sample Number	Company ID
A2780	20211116-03

Sample quantity:1

Characteristics of samples:cell line

Test item:STR

Submitting unit:Fuheng

Detection method:DNA was extracted with Axygen genome extraction kit, amplified with 21-STR amplification scheme, and detected on STR locus and sex gene Amelogenin on ABI 3730XL genetic analyzer.

Detection Result

(1) Basic Inspection Information

Company ID	Multiple alleles	Matched cell line	Cell bank	EV price	Match Descript-ion
20211116-03	Have	A2780	EXPASY	0.90	Basic match

Sample genotype test results

- Polyallelic gene refers to the phenomenon of three or more alleles.
- The cell classification results of the test were good.

(II) Description of Each Sample

- 20211116-03: The DNA typing of the cell strain found a **basic match** in the cell line retrieval. The EXPASY database showed that the cell name was **A2780**, and the cell number corresponded to **CVCL 0134**.**Multiple alleles were found** in this cell line.

Note: The test cell line was compared with STR data of cell lines included in ATCC, DSMZ, JCRB and RIKEN databases. Cell lines not included in these cell banks will not be matched.

(3) Results of Sample Typing

Genotyping results of STR loci and Amelogenin locus in cells						
Loci	STR information of the submitted ce-lls			Cell bank cell STR information		
	Name of the cell submitted for testing: A2780			Cell library cell name: A2780		
	Allele1	Allele2	Allele3	Allele1	Allele2	Allele3
D5S818	10	13		11	12	
D13S317	12	13		12	13	
D7S820	10	10		10	10	
D16S539	11	13		11	13	
VWA	15	16		15	16	
TH01	6	6		6	6	
AMEL	X	X		X	X	
TPOX	8	10		8	10	
CSF1PO	10	11		10	11	
D12S391	19	20				
FGA	19	24				
D2S1338	21	22				
D21S11	28	29				
D18S51	17	17				
D8S1179	15	17				
D3S1358	14	16				
D6S1043	11	17				
PENTAE	10	13				
D19S433	12	12				
PENTAD	8	9				
D1S1656	12	13	15			

Other EXPLANA- TIONS

(1) Classification Scheme and Site Distribution

	Solution 1	Solution 2	Solution 3	Solution 4
1	D3S1358	D8S1179	D19S433	AMEL
2	VWA	D21S11	TH01	D1S1656
3	D7S820	D16S539	D13S317	D5S818
4	CSF1PO	D2S1338	TPOX	D12S391
5	PENTAE	PENTAD	D18S51	FGA
6			D6S1043	

Experimental protocol and site selection

(2) STR Database Comparison

Our company utilizes DSMZ tools for cell line alignment, incorporating STR data from 2,455 cell lines in ATCC, DSMZ, JCRB, and RIKEN databases. If the target cell is not included in these repositories or is a newly established cell line, alignment cannot be performed. Users must then conduct additional comparisons with other databases based on cell typing results.

Issued: 2021-11-19

Sample File	Sample Name	Panel	OS	SQ
33_A05_CellLineAuthentication-1-1117.fsa	A2780	21Plex_STR_Panel_v1.1		

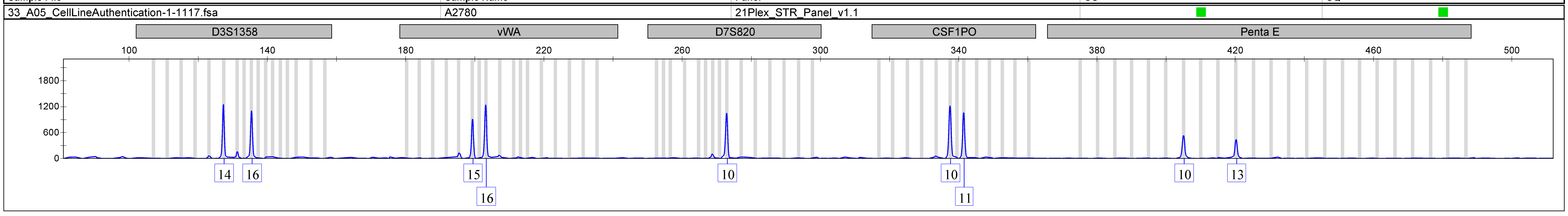

33_A05_CellLineAuthentication-1-1117.fsa	A2780	21Plex_STR_Panel_v1.1		

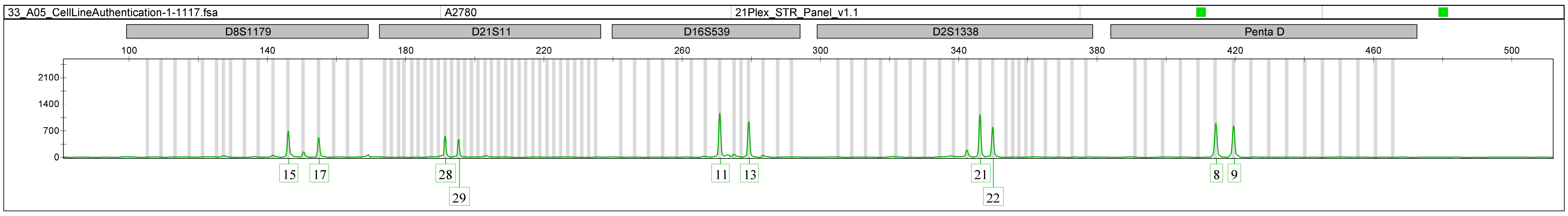

33_A05_CellLineAuthentication-1-1117.fsa	A2780	21Plex_STR_Panel_v1.1		

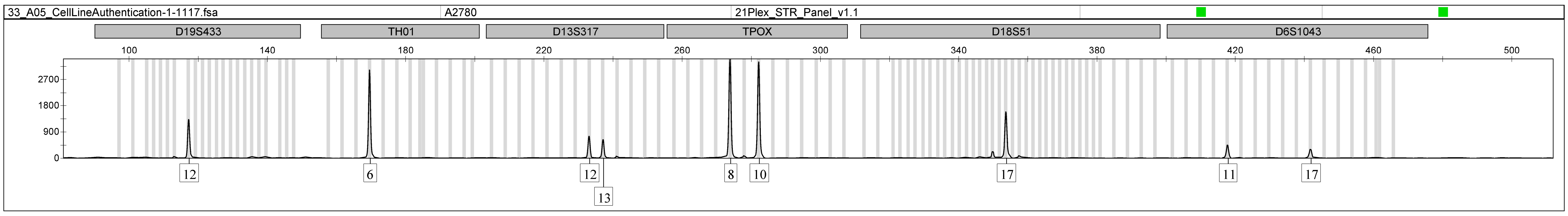

33_A05_CellLineAuthentication-1-1117.fsa	A2780	21Plex_STR_Panel_v1.1		

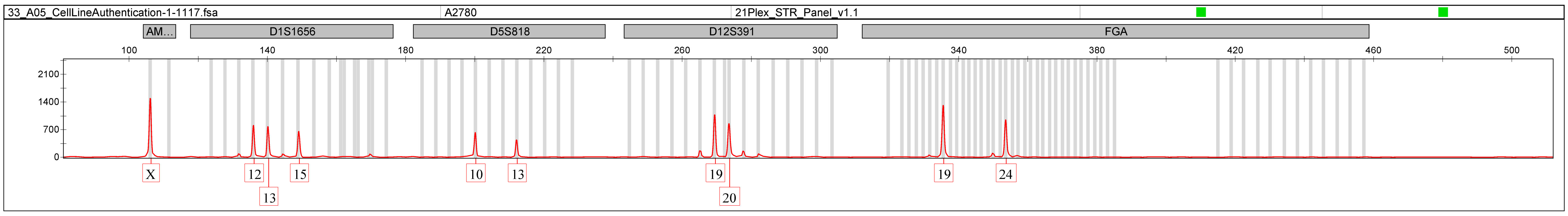

Supplement: A2780_STR.pdf [file KMCO_A_2604899_SM0132.pdf]
