# Supplementary material for: SNX1 inhibits human ovarian cancer progression via regulation of the cell cycle, apoptosis and migration
Source: Mol Cell Oncol. 2025 Dec 31;13(1):2604899. doi: 10.1080/23723556.2025.2604899 (PMC12758353; doi:10.1080/23723556.2025.2604899)
Supplement: Supplementary Figures.docx [file KMCO_A_2604899_SM0133.docx]

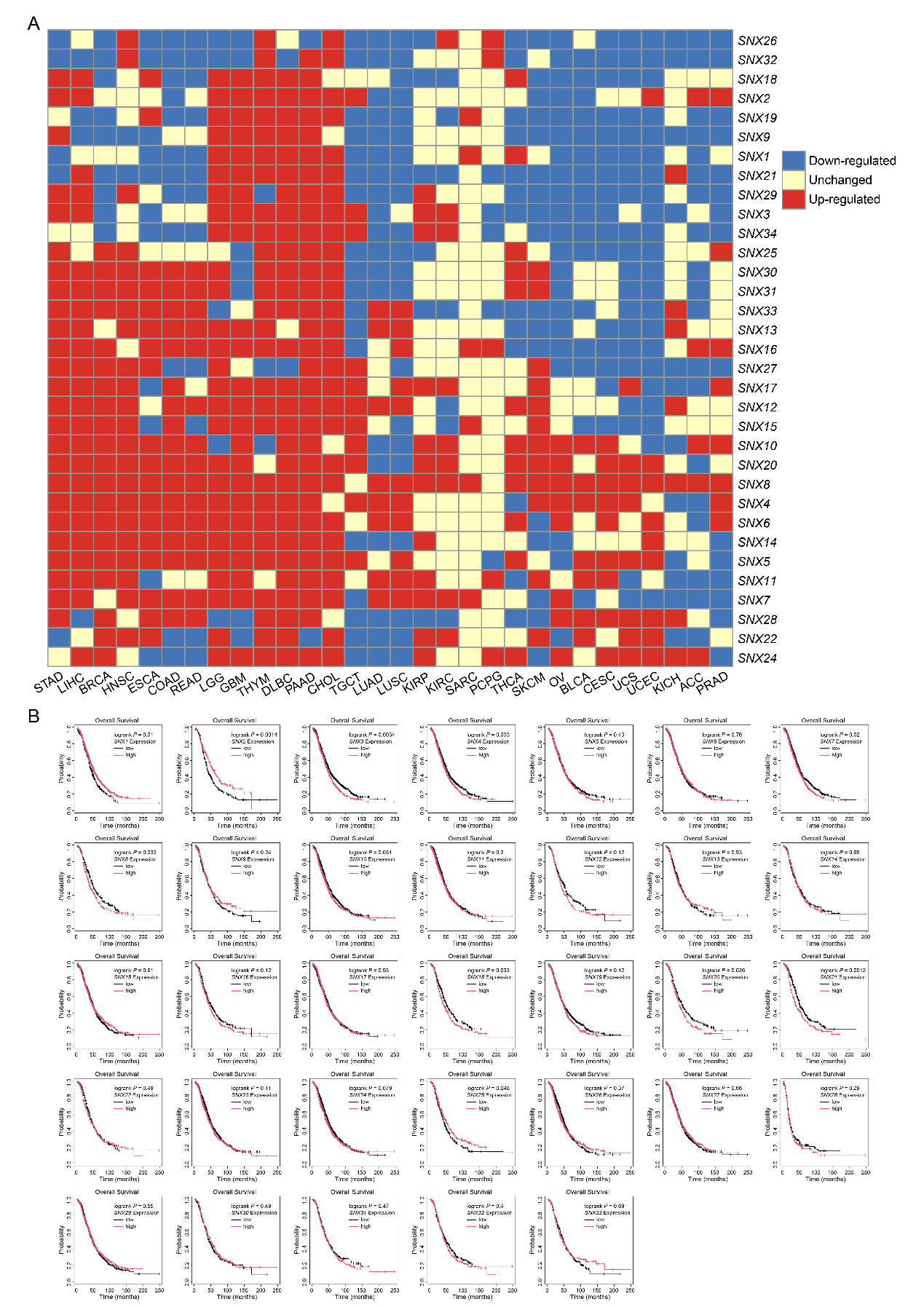


Supplementary Fig. 1 Dysregulation and prognostic value of SNX family members in OV. (A) Pan-cancer analysis of SNX gene expression across tumor types (TCGA) and normal tissues (GTEx). Heatmap colors indicate expression changes of SNX genes in tumors relative to matched normal tissues: red (upregulated), blue (downregulated), and yellow (unchanged). (B) Kaplan-Meier overall survival curve of SNX family in TCGA-OV cohort (log-rank test).


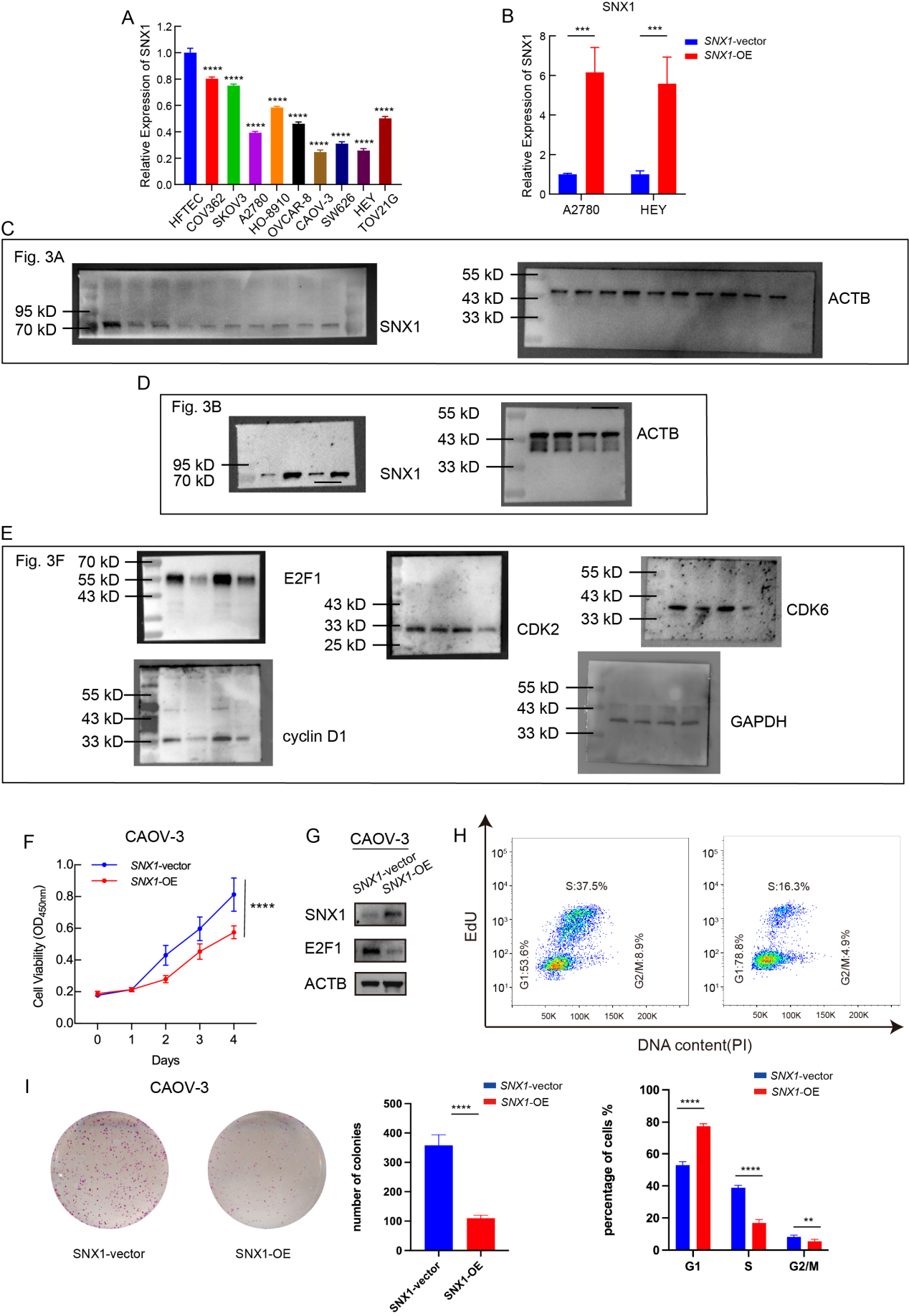


Supplementary Fig. 2 Original western blot images in Fig. 3. (A) Quantitative analysis of western blot images from three independent biological replicates in Fig. 3A (*****P* < 0.0001, n = 3, vs. HFTEC cell lines, Student’s *t*-test). (B) Quantitative analysis of western blot images from three independent biological replicates in Fig. 3B (****P* < 0.001, n = 3, vs. SNX1-low expression groups, Student’s *t*-test). (C-E) Original western blot images used to generate Fig. 3 (A, B, F) and supplementary Fig 2(G). (F) Cell viability assays of SNX1-overexpressing and control groups in CAOV-3 cell detected by CCK-8 assays (****P < 0.0001, n = 5, vs. SNX1-vector groups, two-way ANOVA). (G) Western blot assays of E2F1 and SNX1 protein levels in SNX1-overexpressing and control groups in CAOV-3 cell. (H) EdU/PI Flow cytometric analysis of cell cycle distribution in SNX1-overexpressing and control groups in CAOV-3 cell (***P < 0.001, ****P < 0.0001, n = 3, SNX1-OE groups vs. SNX1-vector groups, two-way ANOVA). (I) Representative images (left) and quantitative analysis (right) of colony formation assays on CAOV-3 cells are shown. Data are presented as the mean number of colonies ± SD from three independent experiments (n=3). Statistical significance was determined by an unpaired two-tailed Student's t-test (****P < 0.0001).


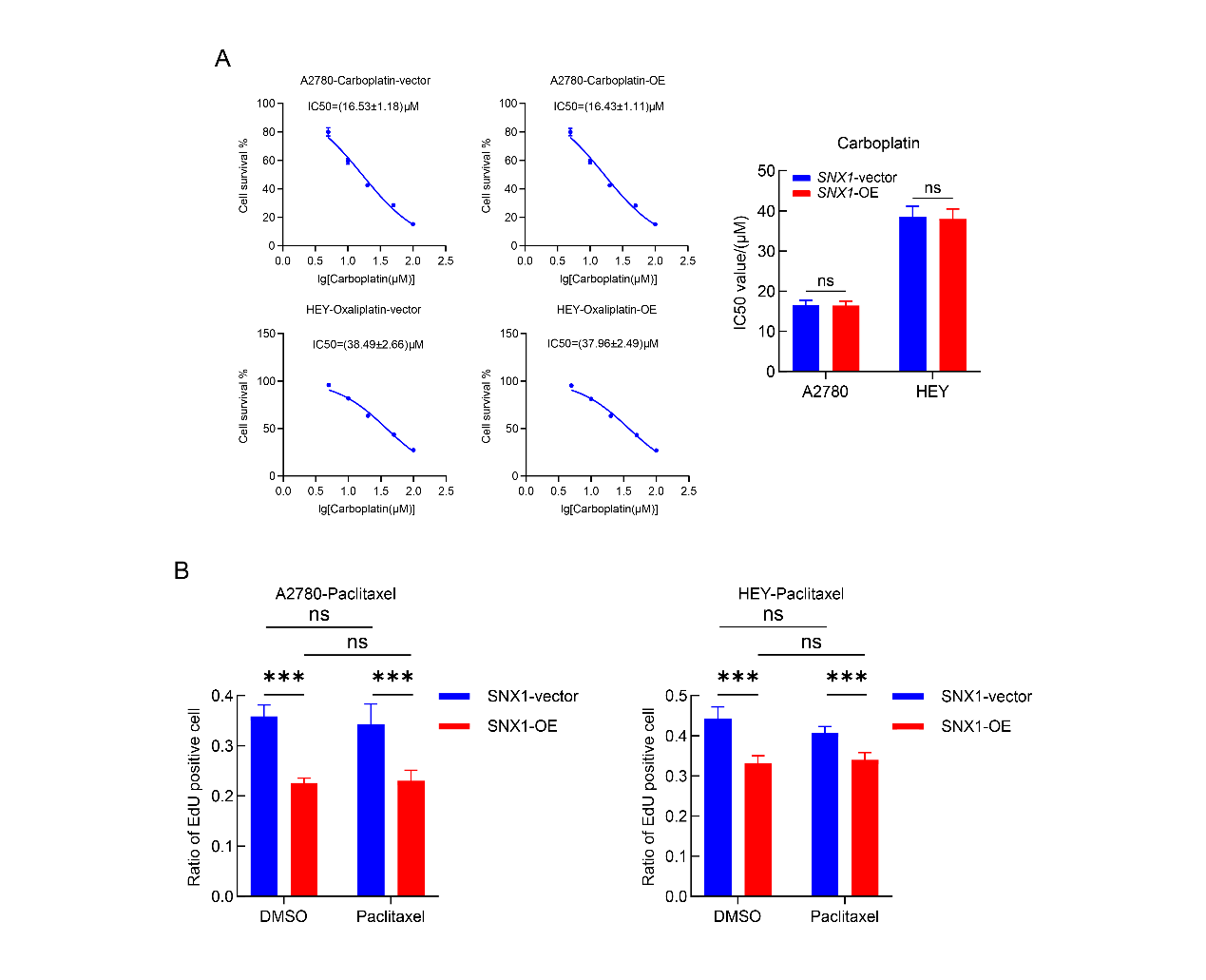


Supplementary Fig. 3 SNX1 enhances sensitivity to paclitaxel in OV cells. (A) The cell survival of SNX1-overexpressing and control groups in A2780 (left) and HEY (right) cells treated with different concentrations of carboplatin was assessed using the CCK-8 assays (n = 5, Student's *t*-test). (B) Quantification of proliferating cells in SNX1-overexpressing and control groups in A2780 (left) and HEY (right) cells treated with different concentrations of paclitaxel (****P* < 0.001, n = 5, two-way ANOVA).
