# Supplementary material for: SNX1 inhibits human ovarian cancer progression via regulation of the cell cycle, apoptosis and migration
Source: Mol Cell Oncol. 2025 Dec 31;13(1):2604899. doi: 10.1080/23723556.2025.2604899 (PMC12758353; doi:10.1080/23723556.2025.2604899)

Assessment of the Quality of Cytogenetic Testing

Cell Line Authentication Service

STR Genotype Test Report

Sample INFORMATION

Sample ID:

Customer Sample Number	Company ID
Caov-3	250927A

Sample quantity:1

Characteristics of samples:cell line

Test item:STR

Detection method: Che lex 100 extracted genomic DNA from cell lines → Si FaSTR 23 plex kit for multiplex PCR → Capillary electrophoresis on ABI 3130 XL genetic analyzer → GeneMapper® ID v 3.2 software for data analysis → Data comparison in ExPASy database.

Detection Result

(1) Basic Inspection Information

Sample Genotype Test Results

Company ID	Multiple alleles	Matched cell line	Cell bank	EV price	Match Descript-ion
250927A	Not have	Caov-3	ExPASy	96.97%	96.97% match

- Polyallelic gene refers to the phenomenon of three or more alleles.

- The cell classification results of the test were good.

(II) Sample Description

250927A: The cell line was identified as a **human cell line**, and its STR typing data in the ExPASy database **matched the Caov-3 cell line 96.97%**.**No polymorphism was found in this cell line, and no cross contamination** was found**.**

Export table

Accession	Name	Nº Markers	Score	Amel	CSF1PO	D2S1338	D3S1358	D5S818	D7S820	D8S1179	D13S317	D16S539	D18S51	D19S433	D21S11	FGA	Penta D	Penta E	TH01	TPOX	vWA
NA	Query	NA	NA		10,13		16	12	10	9,14	12	9	18		30	24			7	10	16,18
CVCL_0201	Caov-3	13	96.97%	X	10,13	16,17	16	12	10	9,14	12	9	18	14,17	30	24	12	11,15	7	8,10	16,18
CVCL_C0UY	NOKT-1	13	58.06%	X	10		16	12	11	13,14	10	9	14		30	23	9	15,20	7	10	14,18
CVCL_0152 *Best*	AsPC-1	13	57.14%	X	10,13	22,23	16	12	12,13	13,15	12	11	18	14	28,30	24	9,12	5,12	7,9.3	8,10	17
CVCL_0152 *Worst*	AsPC-1	13	50.00%	X	11,13	22,23	16	12	12,13	13,15	9,12	11	18	14	28,30	24	9,12	5,12	7,9.3	8,10	17
CVCL_S433	NZM028	13	56.41%	X,Y	11,13	17,18	15,16	11,12	10	13,14	12,14	10,11	13	13,14	29,30	21			6,7	10,11	16,18
CVCL_B859	ESI-053	13	54.05%	X	10		16	11,12	11	10,14	9,12	11	16,18		30,31.2	24	9,11	11,15	6,7	8,11	13,16
CVCL_0950 *Best*	Hs 852.T	13	54.05%	X,Y	13	19	15,16	12	10,11	14,16	9,12	9,11	13.3,15.3	13,15.2	30	18,21	11,13	11,16	9	8,10	18
CVCL_0950 *Worst*	Hs 852.T	13	54.05%	X,Y	13	19	15,16	12	10,11	14,16	9,12	9,11	14,16	13,15.2	30	18,21	11,13	11,16	9	8,10	18
CVCL_B5I1	ISMMSi028-B	13	54.05%	X,Y	10,12		15,16	11	10	14,15	11,12	11	14,18		30,32	24	9,14	12,16	9,9.3	8	16,18
CVCL_B5I2	ISMMSi028-C	13	54.05%	X,Y	10,12		15,16	11	10	14,15	11,12	11	14,18		30,32	24	9,14	12,16	9,9.3	8	16,18
CVCL_0612 *Best*	Kasumi-3	13	54.05%	X,Y	11	18,26	15,16	12	10,11	14	11,12	9,11	14,17	14,15	30	20,24	9,11	16	6,9	8	16,18
CVCL_0612 *Worst*	Kasumi-3	13	50.00%	X,Y	11	18,26	15,16	12	10,11	14	11,12	11	14,17	14,15	30	20,24	9,11	16	6,9	8	16,18
CVCL_1270 *Best*	HCC70	13	52.94%	X	10	23,25	16	12,13	10,11	13	12	9,13	13,16	12.2,15.2	30	24	10	9,16	9	10	13,15
CVCL_1270 *Worst*	HCC70	13	38.89%	X	10,14	25	16,17	13	10,11	13	12	13	13,16	12.2,15.2	30	19.2,24	10	9,16	9	9,10	13,15

- **Note:**The test cell lines were compared against STR reference data from the ExPASy Cell Bank (ExPASy contains STR data for approximately 9,048 human cell lines from databases including ATCC, DSMZ, JCRB, ECACC, and Riken, last updated in August 2025). Cell lines without included STR data could not be compared. According to the ATCC Standards Committee identification criteria (ANSI/ATCC ASN-0002-2022), a match with an EV ≥80% indicates correlation and potential derivation from a common ancestor cell. Matches between 55% and 80% require additional methods to further authenticate their correlation.

(3) Results of Sample Typing

Genotyping results of STR locus and Amelogenin locus in cell 250927A						
Loci	STR information of the submitted ce-lls			Cell bank cell STR information		
	Cell name for submission: Caov-3			Cell bank cell name: Caov-3		
	Allele1	Allele2	Allele3	Allele1	Allele2	Allele3
Amelogenin	X					
D3S1358	16			16		
D5S818	12			12		
D2S1338	16	17				
TPOX	10			8	10	
CSF1PO	10	13		10	13	
Penta D	12					
TH01	7			7		
vWA	16	18		16	18	
D7S820	10			10		
D21S11	30			30		
Penta E	11	15				
D10S1248	15	17				
D8S1179	9	14		9	14	
D1S1656	11					
D18S51	18			18		
D12S391	15	23				
D6S1043	12					
D19S433	14					
D16S539	9			9		
D13S317	12			12		
FGA	24			24		

*Although the table lists the allele data for all loci, the matching algorithm only compares 13 core loci.

Other EXPLANATIONS

(1) Classification Scheme and Site Distribution

Experimental Protocol and Site Selection

	Solution 1	Solution 2	Solution 3	Solution 4
1	D3S1358	Amelogenin	D10S1248	D19S433
2	D5S818	TH01	D8S1179	D16S539
3	D2S1338	vWA	D1S1656	D13S317
4	TPOX	D7S820	D18S51	FGA
5	CSF1PO	D21S11	D12S391	
6	Penta D	Penta E	D6S1043	

(2) STR Database Comparison

STR typing data alignment is performed in the ExPASy database, which contains STR data from 9,048 human cell lines sourced from ATCC, DSMZ, JCRB, and RIKEN (Cellosaurus release 53.0, last updated in August 2025). If the target cell is not included in the ExPASy cell library or is a newly established cell line, STR typing data alignment cannot be performed. Users must then independently cross-reference the cell typing results with other databases.

Applied Biosystems

GeneMapper ID v3.2

250929

Sample Name	Panel	SQO	SQ
CAOV3	SifaSTR 23 plex_v7.0		

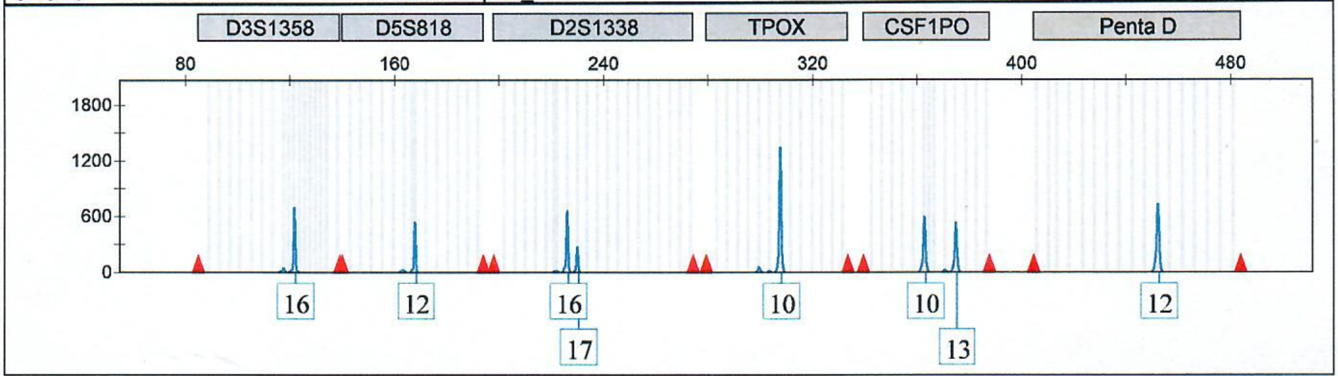

CAOV3	SifaSTR 23 plex_v7.0		

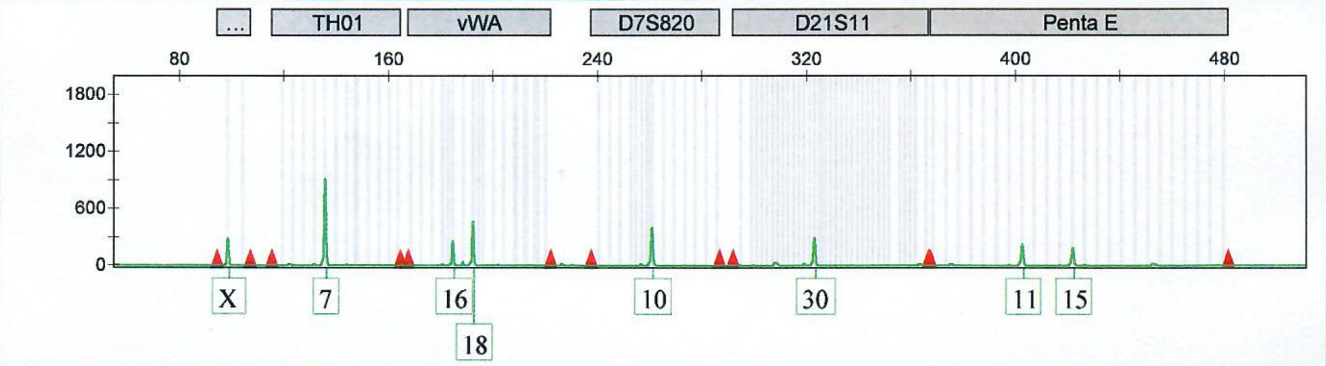

CAOV3	SifaSTR 23 plex_v7.0		

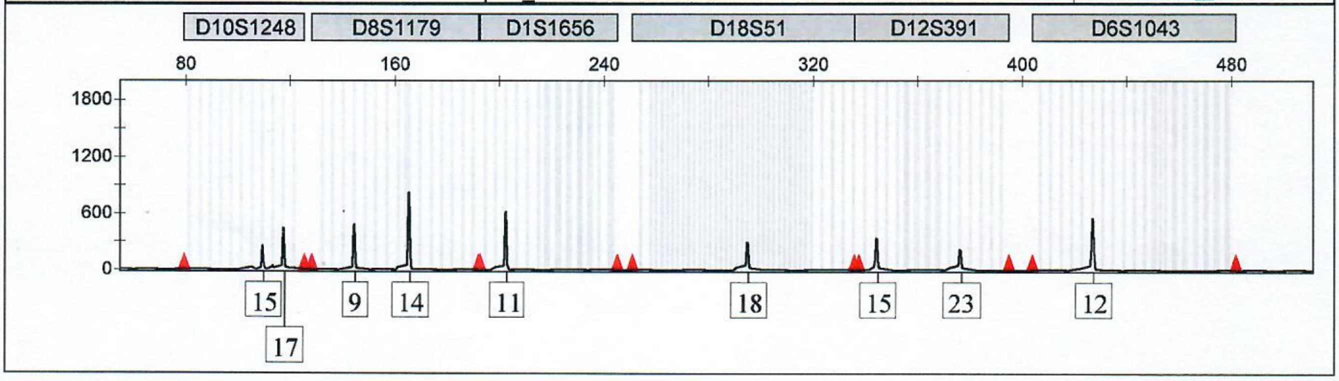

CAOV3	SifaSTR 23 plex_v7.0		

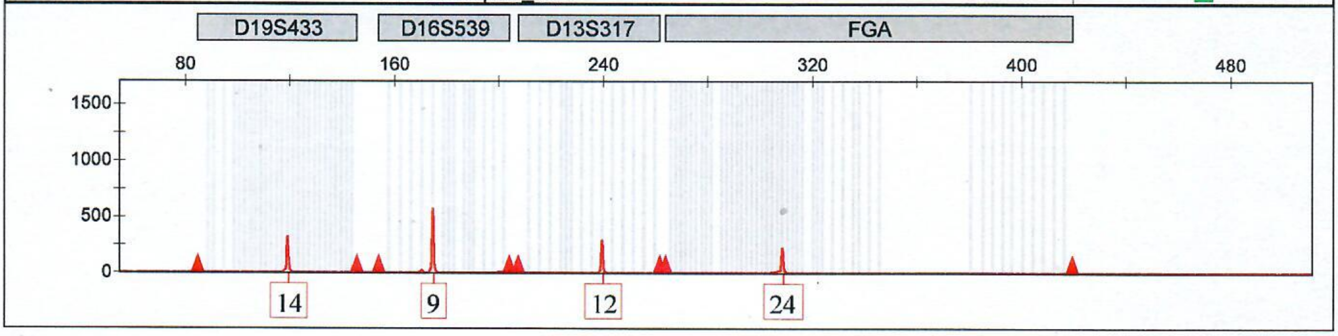

Supplement: CAOV3_STR.pdf [file KMCO_A_2604899_SM0134.pdf]
