# Supplementary material for: SNX1 inhibits human ovarian cancer progression via regulation of the cell cycle, apoptosis and migration
Source: Mol Cell Oncol. 2025 Dec 31;13(1):2604899. doi: 10.1080/23723556.2025.2604899 (PMC12758353; doi:10.1080/23723556.2025.2604899)

Cell genetic quality identification test

Cell Line Authentication Service

STR Genotype Test Report

Sample Information

Sample number:

Customer sample number	Company ID
HEY	20210819

Sample quantity: 1

Sample characteristics: cell line

surveillance project： STR

Sending unit: Shanghai Fuheng Biological Technology Co., LTD

The detection protocol involves: DNA extraction using Axygen's genomic extraction kit, amplification via the 21-STR protocol, and analysis of STR loci and the amelogenin gene (a sex marker) on an ABI 3730XL genetic analyzer.

detection result

(一) Basic inspection information

Company ID	multiple alleles	Match cell lines	cell bank	EV price	Match description

20210819	not have	HEY	ExPASy	1.0	Exact match

Sample genotype test results

- Polyallelic refers to the phenomenon of three or more alleles.
- The cell typing results of this test were good.

(二) Sample Description

- August 19,2021: The DNA profiling of this cell line identified a fully matching cell line in the cell line database. ExPASy database records the cell as HEY, with the cell number CVCL_0297. This test revealed no multi-allelic variants in the identified cell line.

  Note: The STR data of the cell lines to be tested must match those in ATCC, DSMZ (which contains STR data from 2,490 cell strains including ATCC, DSMZ, JCRB, and RIKEN), and ExPASy cell bank (which contains approximately 8,000 human cell STR data from ATCC, DSMZ, JCRB, ECACC, and RIKEN). Cells not included in these databases cannot be matched. According to the ATCC Standards Committee identification criteria (ANSI/ATCC ASN-0002-2022), a match degree of EV ≥80% indicates correlation and potential derivation from a common ancestor cell. For match degrees between 55% and 80%, additional methods are required to further verify their correlation.

(三) Sample genotyping results

**Genotyping results for STR sites and Amelogenin sites in cells**						
Loci	Submit cell STR information			STR information for cell bank cells		
	Cell name for submission: HEY			Cell library cell name: HEY		
	Allele1	Allele2	Allele3	Allele1	Allele2	Allele3
D5S818	11	12		11	12	

D13S317	11	11		11	11	
D7S820	12	12		12	12	
D16S539	8	12		8	12	
VWA	16	17		16	17	
TH01	8	9.3		8	9.3	
AMEL	X	X		X	X	
TPOX	11	11		11	11	
CSF1PO	10	11		10	11	
D12S391	17	22				
FGA	20	21				
D2S1338	24	25				
D21S11	30	30				
D18S51	15	15				
D8S1179	13	13				
D3S1358	16	16				
D6S1043	11	12				
PENTAE	7	13				
D19S433	13	14				
PENTAD	9	13				
D1S1656	12	15				

Other Notes

(一) Classification scheme and site distribution

	Plan 1	Plan 2	Plan 3	Plan 4
1	D3S1358	D8S1179	D19S433	AMEL
2	VWA	D21S11	TH01	D1S1656
3	D7S820	D16S539	D13S317	D5S818
4	CSF1PO	D2S1338	TPOX	D12S391
5	PENTAE	PENTAD	D18S51	FGA
6			D6S1043	

(二) STR database comparison

If the cells to be tested are not included in the international or domestic cell bank, or if the new cell lines are established by themselves, the user will not be able to compare them. The user will have to compare them with the reference data according to the cell typing results, or provide us with assistance in analysis.

Applied Biosystems

GeneMapper ID v3.2

20210819

Sample File	Sample Name	Panel	SQO	OS	SQ
17_A03_CellLineAuthentication-1-0820.fsa	hey	21Plex_STR_Panel_v1.1			

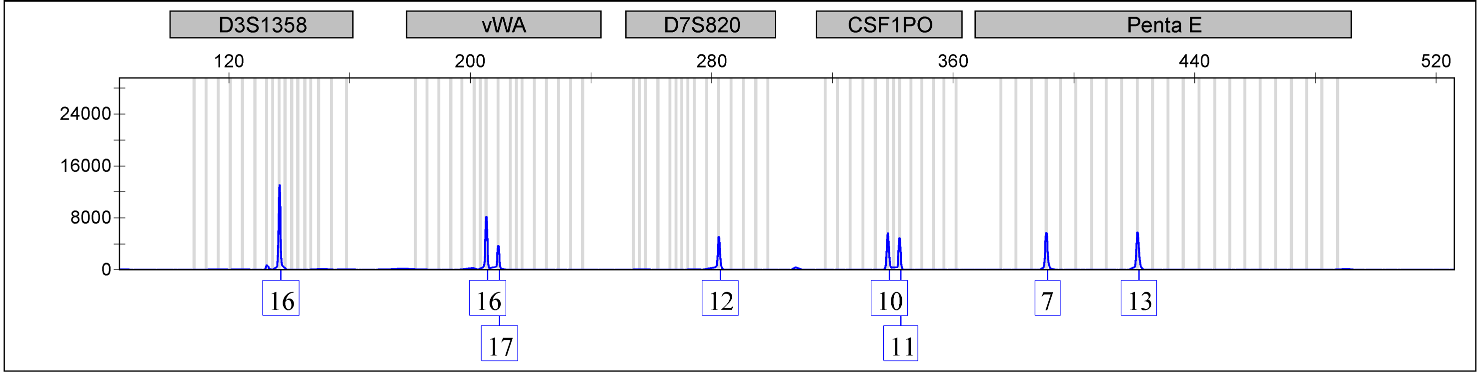

Sample File	Sample Name	Panel	SQO	OS	SQ
17_A03_CellLineAuthentication-1-0820.fsa	hey	21Plex_STR_Panel_v1.1			

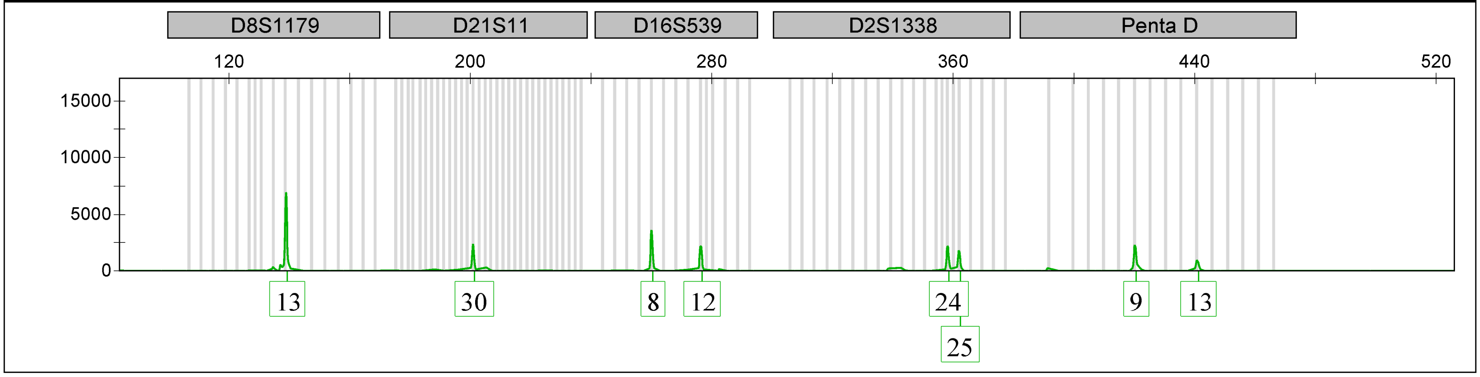

Sample File	Sample Name	Panel	SQO	OS	SQ
17_A03_CellLineAuthentication-1-0820.fsa	hey	21Plex_STR_Panel_v1.1			

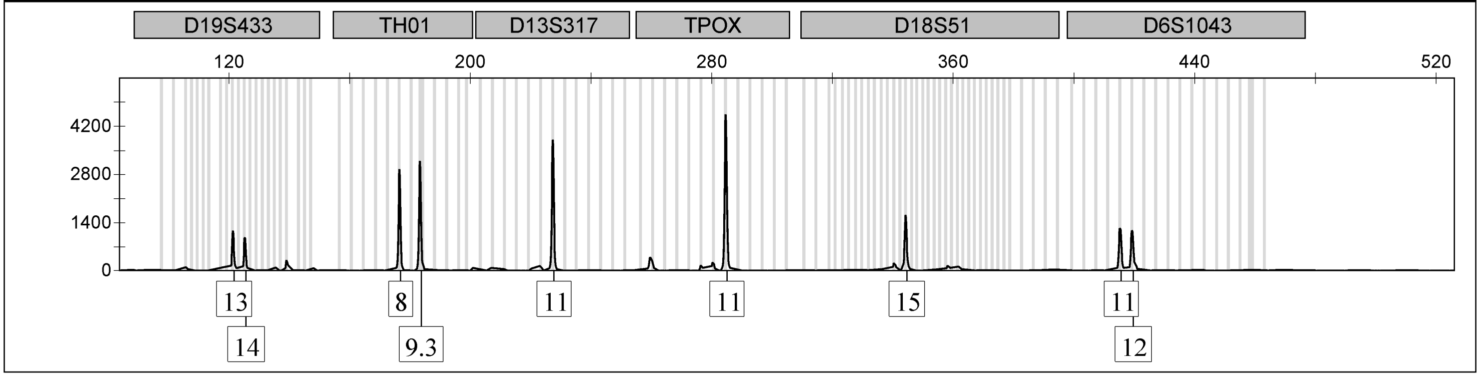

Sample File	Sample Name	Panel	SQO	OS	SQ
17_A03_CellLineAuthentication-1-0820.fsa	hey	21Plex_STR_Panel_v1.1			

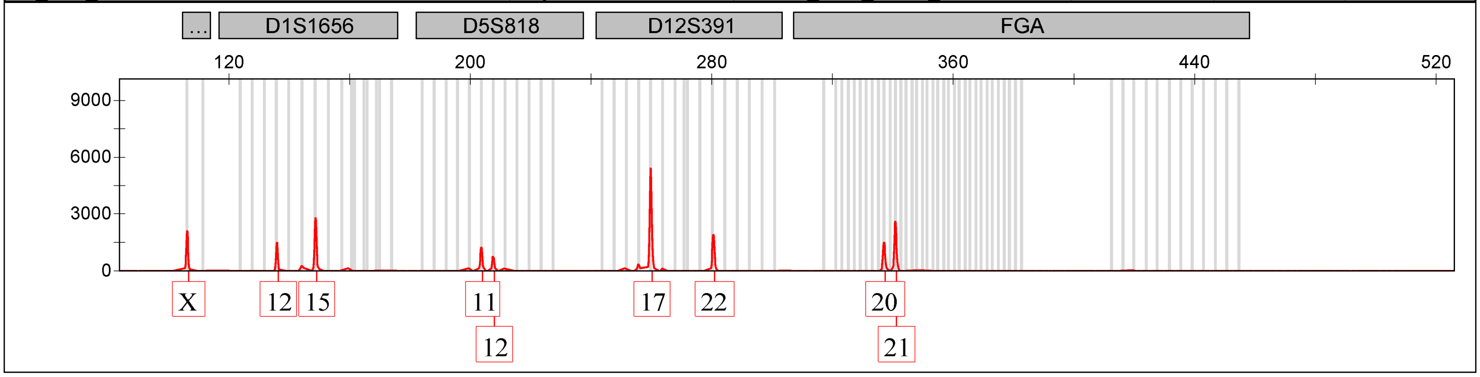

Supplement: HEY STR.pdf [file KMCO_A_2604899_SM0135.pdf]
